# Autopsy on a Case of Prolonged Vomiting

**Published:** 1884-01

**Authors:** H. Wheatley Hart


### Autopsy on a Case of Prolonged Vomiting.

The following case, recently under the care of Mr. Wm. H. Rix, surgeon to the Tunbridge Wells Infirmary, will, I think, be read with interest:

Mrs. E. J——, aged fifty-eight, had suffered more or less for twenty years from this distressing symptom, the character of it being that frequently after food she suffered from a sense of pain and heaviness somewhat above the pit of the stomach, which continued until vomiting ensued. This, however, was not always so, as sometimes during a period of two or three days she would be tolerably free from both pain and sickness. On physical examination of both chest and abdomen, no signs had been discoverable; the urine contained no albumen, and the normal functions, except those of taking food and digestion, had been carried on well. The patient died on July 29th from gradually increasing anæmia and inanition.

At the post mortem the pleuræ were found firmly adherent throughout, but the lungs presented no signs of pressure; the pericardium was also intimately adherent where it met the chest-wall, and also to the whole of the surface of the heart; the left ventricle and aorta were considerably dilated; there were several bronchial glands much enlarged, and in a condition of calcareous degeneration. The mucous membrane of the stomach was fairly normal, with the exception of an area of undue vascularity along the lesser curvature; the organ itself was somewhat shrunken, while the cardiac orifice was excessively contracted, only just admitting the little finger, and that with difficulty; there was, however, no thickened or indurated tissue around the contraction. The œsophagus was enormously dilated above this point of stricture, containing a large quantity of grumous fluid. It occupied a position to the right of the vertebral column, lying in the hollows formed by the angle of the ribs. At the spot where the dilated œsophagus came in contact with the diaphragm it was bent on itself nearly in the form of a right angle, forming a curve as it passed from the right side of the chest to the opening in the diaphragm on the further side of the mesial line. On removing the organ its shape was found to have a remarkable resemblance to an ordinary stomach; it had a gross cubical capacity of twenty-five fluid ounces, and the muscular tissue was found to be very much hypertrophied. The kidneys were slightly granular, but the other organs fairly normal.

The point of interest is that such a partial stricture should have caused so enormous a dilatation above it, and the strong probability is that it was due to the adhesions, existing in the thorax, undergoing gradual contraction, and so causing a deviation of the œsophagus from the direct line at its passage through the diaphragm, and thus was produced a dragging on this structure against the tendinous fibres of the diaphragm, converting a canal, the comfortable patency of which is so necessary to health, into a condition of obliquity and stenosis, varying according to the movements of the diaphragm and the contents of the thoracic cavity.—*H. Wheatley Hart, M. R. C. S., in The Lancet.*

